# Comparison of pulmonary computed tomography angiography findings of COVID-19 cases with and without pulmonary thromboembolism

**DOI:** 10.1097/MD.0000000000041564

**Published:** 2025-09-19

**Authors:** Aida Doulah, Mohsen Hajiani, Yeganeh Karimi, Ali Kabir, Golnaz Moradi, Rayeheh Mohammadi Naeini, Somayeh Jahani Jalaldeh, Maryam Albaji

**Affiliations:** a Department of Internal Medicine, Sina Hospital, Tehran University of Medical Sciences, Tehran, Iran; b Air Pollution and Respiratory Diseases Research Center, Faculty of Medicine, Ahvaz Jundishapur University of Medical Sciences, Ahvaz, Iran; c Tehran Heart Center, Cardiovascular Diseases Research Institute, Tehran University of Medical Sciences, Tehran, Iran; d Minimally Invasive Surgery Research Center, Iran University of Medical Sciences, Tehran, Iran; e Department of Radiology, Sina Hospital, Tehran University of Medical Sciences, Tehran, Iran; f Department of Pulmonary Disease, Sina Hospital, Tehran University of Medical Sciences, Tehran, Iran.

**Keywords:** computed tomography index, COVID-19, pulmonary CT angiography, pulmonary thromboembolism

## Abstract

Since the coronavirus disease 2019 (COVID-19) pandemic, many patients have experienced pulmonary thromboembolism (PTE). In this study, we compared the findings of pulmonary computed tomography angiography (PCTA) in COVID-19 patients with and without PTE. This retrospective study included 168 patients who underwent PCTA. PCTA images were assessed for thromboembolic events, thromboembolism site, parenchymal involvement, severity and stage of involvement, and the computed tomography obstruction index. A total of 168 patients (84 with PTE and 84 without PTE), comprising 103 (61.3%) males with an average age of 59.03 years (95% confidence interval [CI]: 56.81–61.25), were included in the study. No significant differences were observed between the 2 groups in baseline characteristics, except for the mean age. The most common patterns were ground-glass opacities (GGO) (90.70%) and consolidation (44.70%). Age-adjusted logistic regression indicated that PTE patients were 4.5 times more likely to have GGO (*P* = .025, 95% CI: 1.21–16.9). Among patients with PTE, 57.8% had bilateral thromboembolism. Segmental and subsegmental artery involvement was more common in thromboembolic locations. The severity of lung involvement was significantly lower in the PTE group (median, 10; interquartile range, 11) than in the non-PTE group (median, 15; interquartile range, 10; *P* < .001). The average computed tomography obstruction index for patients with PTE was 6.96 (95% CI: 5.48–8.45). Neither intensive care unit admission nor survival rates showed significant differences between the groups (*P* = .618 for intensive care unit admission; *P* = .292 for survival rates). In conclusion, patients with COVID-19 complicated by PTE had higher odds of presenting with GGO. Segmental and subsegmental artery involvement were more common, and PTE did not significantly influence the patient’s outcome.

## 1. Introduction

Coronavirus disease 2019 (COVID-19), caused by Severe Acute Respiratory Syndrome Coronavirus 2, has emerged as a global concern due to its highly contagious nature. The complications associated with COVID-19 encompass a spectrum ranging from acute respiratory distress syndrome and arrhythmias to acute myocardial injury, acute kidney injury, and multi-organ failure.^[1,2]^ Notably, there has been a notable increase in thromboembolic events, such as pulmonary thromboembolism (PTE), venous thromboembolism, and deep vein thrombosis, in COVID-19 patients.^[3–5]^ This surge is attributed to the hypercoagulable state and endothelial injury induced by Severe Acute Respiratory Syndrome Coronavirus 2.^[6]^ The hypercoagulable state in COVID-19 is a consequence of thromboinflammation in the bronchoalveolar compartment, also termed bronchoalveolar hemostasis, as evidenced by the elevated levels of D-dimer, fibrinogen, and fibrinogen degradation products.^[6,7]^ Notably, the presence of PTE in COVID-19 patients is significantly correlated with unfavorable prognoses and an elevated risk of mortality during hospitalization.^[8]^ Furthermore, numerous studies have reported that severe forms of COVID-19 are more prone to complications involving PTE.^[9–11]^ Given the respiratory implications of COVID-19, non-contrast chest computed tomography (CT) has rapidly become a crucial diagnostic and follow-up tool.^[12–14]^ However, worsening clinical presentation, especially in the second week of the disease, is frequently attributed to PTE. Consequently, pulmonary computed tomography angiography (PCTA) is recommended in such cases.^[15]^

PCTA serves a dual purpose of diagnosing PTE and precisely locating the thrombus.^[16]^ Furthermore, it plays a crucial role in assessing the severity of PTE, predicting patient outcomes, and guiding decisions on potential admission to the intensive care unit (ICU) if necessary.^[17]^ Additionally, PCTA provides insights into parenchymal lung involvement in COVID-19, aiding the differentiation between COVID-related and non-COVID-related changes. Despite numerous studies examining PTE, specific PCTA findings in the coexistence of PTE and COVID-19 remain incompletely understood. This study aimed to investigate the correlation between various PCTA findings and PTE in the context of COVID-19 and to determine the diagnostic value of PCTA in identifying PTE.

## 2. Methods

This retrospective study was conducted at the Sina Hospital in Tehran, Iran, between December 2020 and December 2021. Sina Hospital, a referral teaching hospital, has played a significant role as a major center for treating COVID-19 patients. The study was approved by the Research Ethics Committee of Tehran University of Medical Sciences (ethics code IR.TUMS.SINAHOSPITAL.REC.1400.108) and adhered to the principles outlined in the Helsinki Declaration. The inclusion criteria were all hospitalized individuals diagnosed with COVID-19 who underwent PCTA. The exclusion criteria included incomplete medical records and inconclusive pulmonary CT angiography results. The diagnosis of COVID-19 relied on positive real-time polymerase chain reaction results from throat swab samples or negative PCR results coupled with persistent symptoms and characteristic COVID-related changes on chest CT scans. Typical symptoms include fever, dry cough, dyspnea, and myalgia. COVID-related findings on CT scans included bilateral or peripheral ground-glass opacity (GGO) or consolidation. PCTA was performed in patients with suspected PTE symptoms such as worsening dyspnea, chest pain, and elevated serum D-dimer levels. According to our national COVID-19 guidelines, hospitalized patients with moderate to severe/critical lung involvement and sustainable SpO_2_ < 90% despite O_2_ therapy were treated with intravenous dexamethasone 8 mg daily or oral prednisolone 0.5 mg/kg daily for 10 days. Demographic, clinical, and laboratory data were extracted from the medical records.

### 2.1. CT angiography acquisition and image analysis

The PCTA procedure involved injecting patients with a 60 mL iodinated contrast agent at a flow rate of 4 mL/s during maximum inspiration. Two 16-slice multi-detector CT scanners (Philips Ingenuity Flex CT, Philips Healthcare, Best, the Netherlands, and Somatom Emotion, Siemens Healthineers, Erlangen, Germany) at 100 kV, 64 × 0.6 mm, rotation time: 0.37 seconds, average tube current: 207 mA, pitch: 0.9, CTDIvol: 5.88 mGy, and slice thickness: 5 mm with 1 mm reconstruction were used for scanning, covering from the apex to the lung base. A radiologist assessed the images for quality and artifacts using a Picture Archiving and Communication System. PCTA images were scrutinized for thromboembolic events, with locations categorized as proximal, middle, or distal (proximal if the filling defect was detected in the main pulmonary artery trunk or the right or left main pulmonary artery; middle if the filling defect was detected in the lobar arteries or the proximal segmental arteries; and distal if the filling defect was detected in the segmental or subsegmental arteries). Moreover, the distribution of thromboembolic events was reported as right, left, or bilateral. Lung parenchymal involvement was categorized as normal, GGOs, consolidation, crazy paving, and parenchymal bands. The stage of lung parenchymal involvement was determined based on the pattern of parenchymal involvement. GGO pattern, partial crazy paving, and involvement of a few lung lobes were determined as early stage; increases in GGO and crazy paving, and consolidation were detected as progressive; the absorption/late stage was defined as a parenchymal band and regressed opacities. The severity of involvement was reviewed in the semiquantitative scoring system,^[18]^ so each lobe was scored from zero to 5 visually (no involvement scored 0, <5% involvement scored 1, 5–25% scored 2, 26–49% scored 3, 50–75% scored 4, and >75% involvement scored 5). The final score was the summation of all 5 lobes, ranging from 0 to 25. As described by Qandli et al,^[19]^ to determine the pulmonary artery occlusive index, the pulmonary arterial tree was first divided into 3 upper lobes, 2 middle lobes, and lingula, and 5 lower lobes, each consisting of 10 segmental arteries, each of which was scored based on the presence of emboli (one point was given to the presence of emboli in one segmental artery, and the presence of emboli in multiple proximal arteries was given to the number of segmental arteries distal to the proximal emboli). In addition, weighted factors were added based on the degree of vascular obstruction (no thrombus, 0 points; partially occlusive thrombus, 1 point; and totally occlusive thrombus, 2 points). Finally, the CT Obstruction Index was calculated using the following formula:


$$\begin{array}{l} CT\;Obstruction\;Index=\frac{\sum \left(n\cdot d\right)}{40}\times 100 \end{array}$$

where n is the score for proximal thrombi in the pulmonary arterial tree, and *d* is the degree of obstruction.

### 2.2. Statistical analysis

Categorical variables are presented as numbers (percentage), and quantitative variables are presented as means (95% confidence interval [CI]) or medians (interquartile range [IQR]). The 1-sample Kolmogorov–Smirnov test and Shapiro–Wilk test, in addition to the Q-Q plot, were applied to check the normality of the distribution. Chi-square and Fisher exact tests were used to assess the association between categorical variables, while the independent samples *t* test and Mann–Whitney *U* test were applied to compare 2 means. Forward Wald Logistic regression was used to determine independent factors affecting GGO findings in PCTA. All analyses were performed using IBM SPSS Statistics version 26 (Chicago) and STATA software version 17 (StataCorp. LLC, College Station).

## 3. Results

This study, 168 COVID-19 patients were analyzed, comprising 103 (61.3%) males with an average age of 59.03 years (95% CI: 56.81–61.25). Participants were divided into 2 groups: 84 with PTE and 84 without PTE. The baseline characteristics are presented in Table 1. No significant differences were observed between the 2 groups regarding sex, laboratory results, medical history, or symptoms. However, the median age was higher in the PTE group (61.38 years, 95% CI: 58.32–64.43) compared to the non-PTE group (56.69 years, 95% CI: 53.49–59.88, *P* = .036). The PTE group also had a higher proportion of smokers (38.50% vs 13.20%, *P* < .001). Significant differences were noted in prothrombin time, previous COVID-19, hyperlipidemia, liver disease, cancer, and hemoptysis (*P* < .05). Eight of the 9 patients (5.4%) with hemoptysis were in the PTE group (*P* = .034).

**Table 1:** Baseline characteristics of hospitalized COVID-19 patients and differences between patients with and without pulmonary thromboembolism.

Baseline characteristics	Patients with pulmonary thromboembolism n = 84	Patients without pulmonary thromboembolism n = 84	*P*-value
Age (yr); mean (95% CI)	61.38 (58.32, 64.43)	56.69 (53.49, 59.88)	**.036** †
Male; n (%)	55 (65.50)	48 (57)	.267‡
Smoker; n (%)	30 (38.50)	10 (13.20)	**<.001** ‡
Previous history for Covid-19; n (%)	11 (13.40)	2 (2.40)	**.009** ‡
Previous history of hospitalization due to COVID-19; n(%)	17 (20.70)	10 (12)	.132‡
*Laboratory findings*			
Impaired activated partial thromboplastin time (s)*; n(%)	13 (15.70)	17 (20.50)	.420‡
Impaired prothrombin time (s)*; n(%)	55 (67.10)	27 (32.50)	**<.001** ‡
Impaired lactate dehydrogenase*	71 (100)	77 (100)	–
Impaired C-reactive protein*	75 (93.80)	75 (91.50)	.578‡
Impaired D-dimer*	47 (70.10)	43 (46)	.282‡
Anti-neutrophil cytoplasmic antibody (ANCA)	1 (1.19)	–	.999§
*Underling disease, n (%*)			
Cardiovascular disease	21 (25.30)	17 (20.50)	.460‡
Hypertension	30 (96.80)	25 (92.60)	.593§
Pericardial effusion	4 (4.80)	5 (6)	.999§
Cerebrovascular accident	8 (9.50)	3 (3.60)	.119‡
Pulmonary hypertension	4 (4.80)	2 (2.40)	.682§
Pulmonary disease	10 (12)	7 (8.40)	.442‡
Pleural effusion	23 (27.40)	15 (18)	.140‡
Subarachnoid hemorrhage (SAH)	1 (1.19)	–	.999§
Dyslipidemia (DLP)	8 (9.50)	–	**.007** §
Obesity	3 (3.60)	–	.246§
Diabetes mellitus	23 (27.70)	21 (25.30)	.725‡
Tuberculosis	2 (2.40)	–	.497§
Acute kidney injury	–	1 (1.19)	.999§
Chronic kidney disease	–	1 (1.19)	.999§
End-stage renal disease	3 (3.60)	1 (1.19)	.620§
Rheumatoid arthritis	3 (3.60)	2 (2.40)	.999§
Peptic ulcer disease	–	1 (1.19)	.999§
Liver disease	1 (1.19)	8 (9.60)	**.034** §
Cancer	7 (8.40)	–	**.014** §
Hypothyroidism	5 (6)	5 (6)	.999‡
HIV	–	1 (1.19)	.999§
Multiple myeloma (MM)	1 (1.19)	–	.999§
Organ transplantation	1 (1.19)	1 (1.19)	.999§
Craniotomy	1 (1.19)	–	.999§
Psoriasis	–	1 (1.19)	.999§
Psychologic disorders	5 (6)	5 (6)	.999‡
Quadriplegia	1 (1.19)	–	.999§
Reverse inferior vena cava	3 (10)	2 (9)	.999§
Right ventricular strain	1 (1.19)	–	.999§
*Clinical symptoms, n (%*)			
Fever	33 (39.30)	38 (45.80)	.489‡
Cough	44 (53)	52 (62.70)	.209‡
Dyspnea	69 (82)	70 (84.30)	.704‡
Myalgia	26 (31)	26 (31.70)	.916‡
Vomiting	4 (4.80)	3 (3.60)	.999§
Diarrhea	8 (9.50)	6 (7)	.577‡
Hemoptysis	8 (9.50)	1 (1.19)	**.034** §
Gastrointestinal bleeding (GIB)	1 (1.19)	3 (3.60)	.620§
loss of consciousness (LOC)	3 (3.60)	2 (2.40)	.999§

CI = confidence interval, COVID-19 = coronavirus disease 2019. *P* < .05 is considered statistically significant and is indicated in bold.

* aPTT < 40, PT ≤ 13.50, LDH < 233, CRP < 10 and D-dimer ≤ age*10 were considered normal, otherwise abnormal.

† Using an independent sample *t* test.

‡ Using the Chi-square test.

§ Using Fisher exact test.

Table 2 presents the distribution of radiological patterns of lung parenchymal involvement. The most common patterns were GGOs (90.70%) and consolidation (44.70%). Other patterns include crazy paving, parenchymal bands, and atelectasis. GGO was more prevalent in the non-PTE group (*P* = .012). Age-adjusted logistic regression indicated that PTE patients were 4.5 times more likely to have GGO (*P* = .025, 95% CI: 1.21–16.9). Neither the mean age nor sex frequency showed significant differences between patients with GGO and those with consolidation, indicating that there was no association between radiological patterns and age or sex (*P* = .398 for age and GGO, *P* = .215 for sex and GGO, *P* = .114 for age and consolidation, *P* = .678 for sex and consolidation) (Table 3).

**Table 2:** Distribution of radiological patterns of parenchymal involvement among hospitalized COVID-19 patients, categorized by the diagnosis of pulmonary thromboembolism (PTE).

Type of involvement; n (%)	Patients with pulmonary thromboembolism n = 84	Patients without pulmonary thromboembolism n = 84	*P*-value	Total n = 168
Ground-glass opacities	67 (84.80)	79 (96.30)	**.012** *	146 (90.70)
Consolidation	39 (49.40)	33 (40.20)	.244*	72 (44.70)
Crazy paving	4 (5.10)	2 (2.40)	.437†	6 (3.70)
Parenchymal bands	3 (3.80)	3 (3.70)	.999†	6 (3.70)
Atelectasis	2 (2.40)	–	.497†	2 (1.19)

COVID-19 = coronavirus disease 2019. *P* < .05 is considered statistically significant and is indicated in bold.

* Using the Chi-square test.

† Using Fisher exact test.

**Table 3:** The effect of patients’ age and sex on the ground-glass opacities pattern (GGO) and consolidation in hospitalized COVID-19 patients, categorized by diagnosis of pulmonary thromboembolism.

Variables	Patients with pulmonary thromboembolism n = 84	Patients without pulmonary thromboembolism n = 84	Total n = 168
*Age*, *mean (95% CI*)			
Positive report for ground-glass opacities	61.44 (58.06, 64.83)	56.97 (53.66, 60.28)	59.02 (56.65, 61.39)
Negative report for ground-glass opacities	63.91 (53.50, 74.33)	56.33 (9.44, 103.22)	62.40 (53.27, 71.53)
*P*-value*^‡^	0.583	0.942	0.398
*Sex, number with positive report for ground-glass opacities*			
Male	46 (69)	46 (58)	92 (63)
Female	21 (31)	33 (42)	54 (37)
*P*-value	0.321†	0.573†	0.215†
*Age*, *mean (95% CI*)			
Positive report for consolidation	62.33 (57.37, 67.29)	60.24 (55.23, 65.24)	61.37 (57.91, 64.83)
Negative report for consolidation	61.32 (57.09, 65.55)	54.73 (50.43, 59.03)	57.69 (54.63, 60.75)
*P*-value*	.755	.099	.114
*Sex*, *number with positive report for consolidation*			
Male	25 (64)	18 (54.50)	43 (60)
Female	14 (36)	15 (45.50)	29 (40)
*P*-value†	.750	.677	.678

CI = confidence interval, COVID-19 = coronavirus disease 2019.

* Using independent sample *t* test.

† Using the Chi-square test.

^‡^*P* < .05 is considered statistically significant.

This study also examined the severity of lung parenchymal involvement and its correlation with PTE. Most patients in both groups (37 patients with PTE and 49 patients without PTE) were in the early stage of lung parenchymal involvement, with no significant association between lung parenchymal involvement stage and PTE diagnosis (*P* = .803). Twenty-eight (42%) patients with PTE and 30 (36%) without PTE were in the progressive stage. A few patients in both groups (5 patients) were in the absorptive/late stage. However, the quantitative severity of lung parenchymal involvement was significantly lower in the PTE group (median, 10; IQR, 11) than in the non-PTE group (median, 15; IQR, 10; *P* < .001). Among patients with PTE, the median lung parenchymal involvement severity was 10 (IQR:11) and 9.72 (IQR:13) in males and females, respectively. Among the patients without PTE, the median severity was 16 (IQR:8) and 15 (IQR: 10) in the male and female groups, respectively. No significant sex correlations were found with lung involvement severity (*P* = .462 for patients with PTE and *P* = .351 for patients without PTE).

Table 4 presents the distribution of thromboembolisms. Among these patients, 57.8% had bilateral thromboembolism, 30% had thromboembolism localized in the right lung, and 12% had thromboembolism localized in the left lung. Segmental and subsegmental artery involvement was more common in thromboembolic locations.

**Table 4:** Distribution of pulmonary thromboembolism (PTE) in hospitalized COVID-19 patients with a PTE diagnosis, categorized by different lung lobes and pulmonary arteries.

Thromboembolism site, n (%)	RUL n = 17	RML n = 14	RLL n = 51	LUL n = 12	LLL n = 42	PA n = 8
Segmental	7 (41.20)	3 (21.40)	10 (19.60)	3 (25)	12 (28.60)	–
Subsegmental	3 (17.60)	2 (14.30)	16 (31.40)	5 (41.60)	13 (31)	2 (25)
Segmental and subsegmental	4 (23.50)	3 (21.40)	11 (21.60)	2 (16.70)	5 (11.90)	3 (37.50)
Lobar	1 (5.90)	2 (14.30)	3 (5.90)	–	1 (2.40)	–
Lobar and segmental	1 (5.90)	2 (14.30)	7 (13.70)	–	4 (9.50)	1 (12.50)
Other	1 (5.90)	2 (14.30)	4 (7.80)	2 (16.70)	7 (16.60)	2 (25)

COVID-19 = coronavirus disease 2019, LLL = left lower lobe, LUL = left upper lobe, PA = pulmonary artery, RLL = right lower lobe, RML = right middle lobe, RUL = right upper lobe.

The average CT obstruction index for patients with PTE was 6.96 (95% CI: 5.48–8.45). No significant correlation was found between the CT obstruction index and patients’ age (*P* = .447) or sex (*P* = .532). The median time from symptom onset to PTE diagnosis was 12 days (IQR: 9 days), directly correlated with the CT obstruction index (*P* = .028; *R* = 0.245). However, there was no significant correlation between the CT obstruction index and quantitative severity of lung parenchymal involvement (*P* = .369; *R* = 0.102).

Regarding outcome, 58 patients (30 patients with PTE and 28 patients without PTE) required mechanical ventilation, predominantly through invasive methods (26 patients with PE and 18 patients without PTE), with no significant difference between the 2 study groups (*P* = .123). Fifty-three patients were admitted to the ICU (25 with PTE and 28 without), and 124 survived (59 with PTE and 65 without). Neither ICU admission nor survival rates showed significant differences between the groups (*P* = .618 for ICU admission; *P* = .292 for survival rate).

## 4. Discussion

Our findings indicate that individuals with COVID-19 complicated by PTE were more prone to exhibiting GGO patterns in PCTA than those without PTE, with no significant differences observed in consolidation and other patterns between the 2 groups. Both study cohorts predominantly presented in the early stage of lung parenchymal involvement; however, the PTE group demonstrated a lower quantitative severity of lung parenchymal involvement. Additionally, the CT obstruction index was directly correlated with the time interval from symptom onset to PTE diagnosis. Thromboembolisms were predominantly bilaterally localized, with segmental and subsegmental distributions. ICU admission rates or survival outcomes were similar in our study groups.

GGOs are the most prevalent radiological finding in COVID-19 pneumonia, consistent with our observations.^[20]^ Chamorro et al reported GGOs in 32.6% of patients with PTE and COVID-19, with consolidation present in 40.4%.^[21]^ However, the parenchymal involvement pattern did not exhibit a significant association with PTE, a conclusion supported by Badr et al., who found no statistically significant differences in GGOs and consolidation between COVID-19 patients with and without PTE.^[22,23]^ Although we found that patients with PTE were more likely to present GGO, this feature is not a specific PTE characteristic; however, GGO is a diagnostic feature for COVID-19 pneumonia. We considered PTE diagnosis in patients with GGO or other lung involvements with severe presenting symptoms not explained by only parenchymal involvement in COVID-19 patients. Therefore, we recommend evaluating PTE in COVID-19 patients with severe conditions and GGO.

Our study revealed that the majority of patients were in the early stages of parenchymal involvement, with no significant difference between the groups. In concordance with our findings, Stevik et al noted mild-to-moderate lung parenchyma changes in most patients, with no significant association with PTE.^[22]^ Conversely, our results indicating lower severity scores in patients with PTE contrast with studies by Stevik et al, Bompard et al, and Yassin et al, who did not find a significant association between disease severity and PTE incidence.^[22,24,25]^ In contrast, Ooi et al reported an increased prevalence of PTE in patients with moderate-to-severe COVID-19.^[26]^ Additionally, Yassin et al reported a median time of 12 days from disease onset to the incidence of PTE.^[24]^ The duration was 9.7 days in a study performed by Garcia-Olivé et al^[27]^ and 12 days in a study performed by Grillet et al,^[28]^ consistent with our results. The correlation between the time interval from symptom onset to the diagnosis of PTE and the CT obstruction index, as noted in our study, emphasizes the progressive nature of COVID-19, consistent with the study by Yassin et al.^[24]^ Similar to our study, previous investigations have localized PTE in segmental and subsegmental arteries.^[22,23,26,29,30]^ We also observed a higher proportion of PTE localized in the right and left lower lobes, which can be explained by gravity-dependent blood flow. Regarding our findings and previous studies, PTE is more likely to be localized in smaller vessels (segmental and subsegmental arteries) in lower lobes in patients with COVID-19, though it can involve main arteries and upper lobes.

Our findings revealed no significant differences in mechanical ventilation, ICU admission, and mortality between the study groups, consistent with previous studies.^[23,31–34]^ Conversely, contrasting evidence suggests higher ICU admission and mortality rates in PTE patients.^[30,35,36]^ Noteworthy, the time interval from symptoms onset to diagnosis and treatment of PTE, adequate anticoagulation therapy, and demographic characteristics play a crucial role in patient outcomes, particularly in mortality.^[37]^ However, our study was limited in evaluating related variables. Further prospective studies evaluating associated factors should be considered for the effect of PTE on the patient’s outcomes. Nevertheless, we showed that patients with PTE can have similar outcomes to patients without PTE with timely diagnosis and interventions.

This study thoroughly explains PCTA findings in patients with COVID-19 who experience complications from PTE. Noteworthy strengths include the substantial sample size and the inclusion of a control group. However, this study has some limitations. The retrospective design, single-center data collection, and absence of long-term follow-up constrain the generalizability of our findings. To address these limitations, we recommend that future studies adopt a prospective multicenter approach, incorporate long-term follow-up, consider thromboprophylaxis, and evaluate biomarkers for a more comprehensive analysis of this topic.

## 5. Conclusion

In conclusion, patients with COVID-19 complicated by PTE have higher odds of presenting with GGOs and segmental/subsegmental thrombosis. A notable correlation was also identified between the duration from symptom onset to PTE diagnosis and the CT obstruction index. Importantly, this investigation underscores that PTE does not significantly influence patient outcomes.

## Acknowledgments

We would like to thank all patients who agreed to use their data in this study.

## Author contributions

**Conceptualization:** Aida Doulah, Mohsen Hajiani, Maryam Albaji.

**Data curation:** Ali Kabir.

**Formal analysis:** Ali Kabir.

**Resources:** Aida Doulah, Golnaz Moradi, Rayeheh Mohammadi Naeini, Somayeh Jahani Jalaldeh.

**Supervision:** Maryam Albaji.

**Validation:** Ali Kabir.

**Writing – original draft:** Yeganeh Karimi.

**Writing – review & editing:** Aida Doulah, Yeganeh Karimi, Ali Kabir.
